# Phytochemical Content, Antibacterial and Antioxidant Potential of Endemic Plant *Anabasis aretioïdes *Coss. & Moq. (Chenopodiaceae)

**DOI:** 10.1155/2020/6152932

**Published:** 2020-02-01

**Authors:** Souad Senhaji, Fatima Lamchouri, Hamid Toufik

**Affiliations:** Laboratory of Materials, Natural Substances, Environment and Modeling (LMSNEM), Polydisciplinary Faculty of Taza, Sidi Mohamed Ben Abdellah University of Fez, B.P. 1223, Taza-Gare, Taza, Morocco

## Abstract

This study aims to investigate the biological activities of endemic plant *Anabasis aretioïdes* harvested in eastern Morocco. Various extracts were prepared from aerial part by aqueous and organic extraction using a Soxhlet and cold maceration. Preliminary phytochemical analysis was carried out on the powder and on the different extracts by standard phytochemical tests, and was confirmed by a quantitative analysis based on the determination of total polyphenols and cathechic tannins. Antioxidant activity was evaluated *in vitro* by five methods: H_2_O_2_, DPPH, ABTS, FRAP, and RP, and the antibacterial activity was carried out by disc diffusion method and the determination of MIC and MBC. Phytochemical screening revealed the presence of cathechic tannins, saponins, and sterols and quantitative analysis showed that Ethyl acetate extract presented the high level of phenolic and cathechic tannins contents (46.79 ± 0.75 *µ*g GAE/mg E and 46.46 ± 0.67 *µ*g CE/mg E). A highest hydrogen peroxide activity was observed in aqueous macerated extract (7.84 ± 0.44%) and the macerated methanol extract has the highest rates for the other four antioxidant activity tests: It was able to reduce DPPH with an IC_50_ of 52.91 ± 0.24 *µ*g/ml, the highest ABTS•+ radical scavenging capacity (48.99 ± 1.316 *µ*g TE/mg E), it showed also the highest antioxidant activity by the FRAP and reducing power test (99.73 ± 3.570 *µ*g TE/mg E and 72.176 ± 0.540 *µ*g AAE/mg E). Antibacterial screening showed that the maximum zone of inhibition was noted for ethyl acetate extract against *Staphylococcus aureus* (13.5 mm). The lowest MIC value was obtained with methanolic and macerated methanolic extracts against *Protéus mirabilis *strain (MIC = 3.125 mg/ml). Principal component analysis showed that the four methods ABTS, DPPH, FRAP, and RP are highly correlated and a correlation between the antioxidant activity and the total phenolic contents of the extracts indicated that phenolic compounds were the dominant contributors to the antioxidant activity of the plant.

## 1. Introduction

The continued use of traditional medicine is attributable not only to cultural and poverty reasons but also to the ineffectiveness of many existing medicines [1]. The lack of effective remedies and the resistance created by current antibiotic pathogens, as well as oxidative stress new therapeutic agents from plants [2–5]. Indeed, many studies have shown that plants possess antioxidant properties due largely to their phenolic compounds [6]. These compounds play an important role in human health with their pharmacological activities, such as anti-inflammatory, anti-allergic, antimicrobial, antiviral, anti-cancer, cardioprotective, and vasodilatory activities [7, 8]. In addition, they can prevent oxidative modification by neutralizing free radicals, oxygen scavenging, or decomposing peroxides through their antioxidant activities [9]. To this end, new natural antioxidant molecules are being researched in medicinal plants for the role they can play in the prevention of diseases such as cancer, diabetes, hypertension, and Alzheimer's disease by combating oxidative stress and its associated pathologies. Endemic plants can be a source of new active ingredients to fight antibiotic resistance as well. Also, these plants have been used for a long time to fight against all kinds of infections, and some pathogenic microbial species, are less sensitive to antibiotics and develop multiple resistances. Consequently, in the face of the appearance of resistant forms of several bacteria to certain antibiotics, the search for new active molecules with a broad spectrum of action has become a necessity [10]. One of the strategies for this research is to explore plants used in traditional medicine, that have been used for a long time to fight against cutaneous, respiratory, and viral infections [11]. Morocco has an important floristic richness estimated at 4500 taxa with 920 genera and 130 families [12], which can be exploited to find new original active ingredients. It occupies the first place among the countries of the South of the Mediterranean for its richness in endemic plants [12]. Among the total number of endemics estimated at 1471, there are 807 taxa located in Moroccan territory and 664 endemics that extend beyond the geographical framework of Morocco such as macaronesian endemics, betico-rifain endemics, and Moroccan–algerian endemics [13]. There are several endemic Moroccan–algerian plants that have been studied very rarely, but have real pharmacological properties. This is the case of *Anabasis aretioïdes*, (Syn. *Fredolia aretioïdes* Coss. & Dur.) of the family *Chenopodiaceae*, endemic plant of Morocco and Algeria [14].

In Morocco, *Anabasis aretioïdes *Coss. & Moq. is known by several vernacular names: "Sejra li ma idihach rih, Sejra li ma ihezhas rih, Akennud, and by Dega and in Algeria under the name of chou de Bou Hamama. This plant is widely used in traditional medicine, the leaves and roots are prepared as an infusion or decoction as anti-rheumatic, diuretic, hypoglycemic, and poison antidote in Morocco and Algeria. The bark of the root is used as firewood [14–16]. These therapeutic properties of *Anabasis aretioïdes* Coss. & Moq. are related to its richness in chemical compounds possessing biological properties *in vitro*: antimitotic, according to the urchin egg test with an IC_50_ = 1.790 g/l [17] and antioxidant activity [18, 19].

The bibliographic studies did not show any reference to previous work on the free radical scavenging and/or antioxidants and antibacterial properties of *Anabasis aretioïdes *Coss. & Moq. that grows in eastern Morocco, but others works have been carried out on *Anabasis aretioïdes* collected in Algeria for both antioxidant activity and phenolic compound determination. Therefore, the objective of this study was to conduct a phytochemical study of the aerial part of *Anabasis aretioïdes* Coss. & Moq. in the Figuig region (Morocco), which involves the preparation of several aqueous (decoction, infusion, maceration) extracts, organic by Soxhlet using four different solvents with increasing polarity namely (methanol, ethyl acetate, chloroform, and petroleum ether), and by cold maceration with methanol for screening test and determination of total polyphenols and cathechic tanins. The pharmacological study consisted of an evaluation of *in vitro* antioxidant and anti-radical activities by five analytical methods: H_2_O_2_ (hydrogen peroxide scavenging), DPPH (scavenging activity method), ABTS (2,2′-azinobis-3 ethylbenzothiazoline-6-sulfonic acid), FRAP (Ferric reducing/antioxidant power), reducing power method (RP), and the determination of *in vitro* antibacterial activity by the disc diffusion method in an agar medium then the determination of the minimum inhibitory (MIC) and bactericidal (MBC) concentrations of the strains that showed sensitivity to the tested extracts.

## 2. Materials and Methods

### 2.1. Plant Material

*Anabasis aretioïdes *Coss. & Moq. was collected during the month of September 2016 from the region of Figuig (Morocco) (GPS: North 32°06′32″, Wes 001°13′42″, Altitude: 898 m) and was identified by Dr. Abdelmajid khabach at the Laboratory Materials, Natural Substances, Environment & Modelling (LMSNEM), Polydisciplinary Faculty of Taza, Sidi Mohamed Ben Abdellah University of Fez, Morocco. The aerial part was removed from the sand, then dried in a dry and ventilated place away from the light, and then preserved. A herbarium number SF2016/01 has been prepared and archived in our MSNEM laboratory at the Polydisciplinary Faculty of Taza.

### 2.2. Phytochemical Study of *Anabasis aretioïdes* Coss. & Moq.

#### 2.2.1. Preparation of Different Extracts of *Anabasis aretioïdes* Coss. & Moq.

During this work, we used various hot and cold extraction techniques using distilled water to prepare aqueous extracts and organic solvents with different polarity (methanol, ethyl acetate, chloroform, and petroleum ether) for organic extracts.

*(1) Preparation of Aqueous Extracts. Decocted:* 20 g of the aerial part of *Anabasis aretioïdes* Coss. & Moq. was boiled with 200 ml of distilled water, using a heating mantle for 20 min.

*Infused:* 20 g of the aerial part of *Anabasis aretioïdes *Coss. & Moq. was put in a beaker with 200 ml of hot distilled water for 30 min.

*Macerated:* 20 g of the aerial part of *Anabasis aretioïdes* Coss. & Moq. was put in a beaker with 200 ml of cold distilled water for 24 hours.

The three prepared extracts were frozen at −80°C, lyophilized, weighed and then stored at 4°C.

*(2) Preparation of Organic Extracts. *

1. *Soxhlet Extraction.* Four solvents with different polarity (methanol, ethyl acetate, chloroform, and petroleum ether) were used to prepare the organic extracts. (20 g) of the aerial part of *Anabasis aretioïdes* Coss. & Moq. was extracted into 200 ml of solvents using a soxhlet extractor for 6 h. Then, the mixture was thoroughly filtered using a filter paper and the solvent was removed using a rotary evaporated (Buchi R-210). The extracts obtained were weighed and then stored at 4°C.
2. *Cold Maceration by Methanol.* 20 g of the plant material was macerated in 200 ml of methanol for 48 hours. After filtration, the methanolic solution of the aerial part of *Anabasis aretioïdes* Coss. & Moq. was thoroughly filtered using a filter paper and the solvent was removed using a rotary evaporated (Buchi R-210). The dry residue obtained was then weighed and stored at 4°C until further use.

#### 2.2.2. Phytochemical Screening

The search for the major families of secondary metabolites was made in the dried plant powder and in the aqueous and organic extracts of the aerial part of *Anabasis aretioïdes* Coss. & Moq. using a qualitative analysis based on coloring reactions and/or precipitation. Flavonoids were searched by the cyanidin reaction [20]. For the characterization of catechic and gallic tannins, we used ferric chloride [21]. The identification of saponins was based on their ability to form a mousse [22], the sterols were searched by the reaction of Liebermann [20], and we characterized the alkaloids by Dragendorff reagent (potassium iodobismuthate) and Valser-Mayer reagent (potassium tetra-iodomercurate) [23]. The presence of anthracenosides was searched by the Borntraeger reaction [24],the KOH reagent was used for the detection of anthraquinones [25], and the presence of free quinones is confirmed by adding a few drops of NaOH [26]. The dosage of secondary metabolites of *Anabasis aretioïdes* Coss. & Moq. was conducted according to the results of phytochemical screening tests. Thus, we measured total polyphenols and catechic tannins.

#### 2.2.3. Determination of Phenolic Contents

*Determination of Total Polyphenols.* The total polyphenol content was determined using the Folin–Ciocalteu reagent, using the method of Lister and Wilson [27]. 0.5 ml of each sample was introduced into test tubes, (2.5 ml) of Folin–Ciocalteu's reagent, previously diluted with water (1:10 v/v) and 4 ml (7.5%, (m/v)) of Na_2_CO_3_ and kept in a water bath (50°C) for 30 minutes. The absorbance of all samples was measured at 765 nm using a spectrophotometer (UviLine 9100-94000UV/Vis). The results are expressed as micrograms gallic acid equivalent per milligram of extract (*µ*g GAE/mg).

*Determination of Cathechic Tannins.* The quantities of cathechic tannins were estimated using the vanillin method in an acid medium [28]. A volume of 50 *µ*l of the extract was added to 1.5 ml of the vanillin/methanol solution (4%, m/v) and then mixed using a vortex. Then, 750 *µ*l of concentrated hydrochloric acid (HCl) was added and allowed to react at room temperature for 20 min. Absorbance at 500 nm was measured using a spectrophotometer (UviLine 9100-94000UV/Vis). The concentration of tannins was expressed in micrograms equivalent of the catechin per milligram of extract (*µ*g CE)/mg) from the calibration curve.

### 2.3. Determination of Antioxidant Activity

The *in vitro* antioxidant activity of our extracts was estimated by five tests: H_2_O_2_ (hydrogen peroxide scavenging), DPPH (scavenging activity method), ABTS (trolox equivalent antioxidant capacity assay), FRAP (Ferric reducing/antioxidant power), and reducing power assay (RP).

#### 2.3.1. Hydrogen Peroxide Scavenging Activity

The ability of the plant extract to remove hydrogen peroxide was determined according to the method of Ruch and its collaborators [29]. A solution of 40 mM H_2_O_2_ was prepared in phosphate buffer saline (PBS) (50 mM, pH 7.4). 1 ml of sample (100 *μ*g/ml) soluble in distilled water was added to 0.6 ml of a solution of hydrogen peroxide in PBS. The absorbance was measured at 230 nm, after 10 minutes, against a blank solution containing extracts and PBS without hydrogen peroxide.

(1) $$\begin{array}{c} \text{Percentage}\;\text{of}\;\text{hydrogen}\;\text{peroxide}\;\text{scavenging}=\left[\left(A_{i}-A_{t}\right)/A_{i}\right]*100, \end{array}$$

*A*_t_= absorbance of the sample *A*_i_ = absorbance of control

where the control is the phosphate buffer with H_2_O_2_.

#### 2.3.2. DPPH Radical Scavenging Assay

The method used to evaluate the anti-radical activity of the various extracts of *Anabasis aretioïds *Coss. & Moq. is that described by Sharma and Bhat [30]. It consists in preparing a methanolic solution of DPPH at 200 μmol/L. Then, in dry tubes, we introduced 3 ml of extract and then we added 1ml of DPPH solution. After agitation, the tubes were incubated at 30°C for 30 min in the dark. The results were read by measuring the absorbance at 517 nm by a spectrophotometer (UviLine 9100-94000UV/Vis).

The control consists of 3 ml of methanol and 1 ml of DPPH solution. The positive control is represented by a solution of standard antioxidants, ascorbic acid, Trolox, and BHT, whose absorbance is measured under the same conditions as the sample tested.

The percentage inhibition of DPPH is calculated according to the following formula:

(2) $$\begin{array}{c} \%\text{Inhibition}=\left[\left(\text{Abs}\;\text{control}-\text{Abs}\;\text{test}\right)/\text{Abscontrol}\right]\times 100. \end{array}$$

#### 2.3.3. ABTS Radical Scavenging Assay

The ABTS assay was realized according to the method of Re and its collaborators [31]. An ABTS solution at a concentration of 7 mM is prepared in demineralized water, which we mixed with a solution of potassium persulfate with a concentration of 2.45 mM with a ratio of 1 : 0.5. The mixture was allowed to stand for 12–16 h at 30°C in the dark. The ABTS^+^ solution was then diluted with ethanol until (0.70 ± 0.02) absorbance at 734 nm.

30 *μ*l of samples diluted in ethanol were mixed with 3 ml of the radical solution, the mixtures were allowed to stand for 1 min and their absorbance's were measured at 734 nm. A Trolox calibration curve was prepared in the range 5–400 *μ*g/ml and the antioxidant activity is expressed in Trolox equivalent capacity (*μ*g TE/mg E),

#### 2.3.4. Ferric Reducing/Antioxidant Power FRAP Assay

The FRAP test was performed using the Benzie and strain method [32]. The test consists of mixing, 3 ml of FRAP solution +100 *µ*L of each extract or Trolox. First, we prepared an acetic acid/sodium acetate buffer solution at 300 mM at pH = 3.6. Then, the TPTZ reagent at 10 mM, diluted in HCl at 40 mM, and the FeCl_3_ reagent at 20 mM are prepared extemporaneously. Finally, the FRAP solution is made by mixing 2.5 ml of TPTZ solution, 2.5 ml of FeCl_3_ solution and 25 ml of sodium acetate buffer solution. Absorbance is measured at 595 nm. A Trolox calibration curve was prepared in the range 5–400 *μ*g/ml and the antioxidant activity is expressed in Trolox equivalents capacity (*μ*g TE/mgE).

#### 2.3.5. Reducing Power Assay

The reducing power was quantified by the method described by Oyaizu [33]. 1 ml of the sample or ascorbic acid, 2.5 ml of phosphate buffer (0.2 M, pH 6.6), and 2.5 ml of ferricyanide of potassium (1%) were incubated at 50°C for 20 min. After cooling, the reaction was terminated by adding 2.5 ml of the trichloroacetic acid (TCA) solution (10%) and centrifuged at 3000 rpm for 10 min. The supernatant (2.5 ml) was mixed with 2.5 ml of distilled water and 0.5 ml of 0.1% ferric chloride. The absorbance of the reaction mixture was read at 700 nm. Ascorbic acid was used as a positive control.

### 2.4. Antibacterial Activity

The antibacterial activity of the organic extracts of the aerial part of *Anabasis aretioïdes* Coss. & Moq. was evaluated using six bacteria strains, three Gram positive, namely: *Staphylococcus aureus* CECT976, *Bacillus subtilis* DSM6633, *Listeria innocua* CECT 4030 and three Gram negative: *Escherichia coli* K12, *Protéus mirabilis*, *Pseudomonas aeruginosa *CECT118. The study of the antibacterial activity is carried out by different and complementary techniques: disc diffusion method in an agar medium then the determination of minimum inhibitory concentrations (MIC) and bactericidal (MBC).

#### 2.4.1. Disc Diffusion Method

Methanol, macerated methanol, ethyl acetate, chloroform, and petroleum ether organic extracts, are solubilized in 10% dimethyl sulfoxide (DMSO).

For each organic extract, we prepared the concentrations of 40 mg/ml, 80 mg/ml, and 100 mg/ml, and then the different solutions prepared were sterilized by filtration on 0.45 *µ*m filters.

Petri dishes containing Mueller Hinton agar are swabbed with a suspension (about 10^8^ CFU/ml) on a Mac Farland scale, which comes from a young culture of bacteria. After drying the dishes, the discs (6 mm in diameter), soaked in 40 mg/ml, 80 mg/ml, and 100 mg/ml of organic extracts were deposited on the surface of an agar medium (Mueller–Hinton). After incubation for 24 h at 37°C, the antibacterial activity was evaluated by measuring the zone of inhibition against the tested organisms. All tests were repeated three times. Negative controls were performed by DMSO at 10%, while tetracycline and Amikacin were used as positive controls [34].

#### 2.4.2. Determination of the Minimum Inhibitory Concentration (MIC)

It is a method that allows us to determine the smallest concentration of extracts sufficient to inhibit the growth of a bacterial strain (MIC).

MICs were determined in liquid medium (Mueller–Hinton) in sterile plastic microplates containing 96 wells. The extracts were dissolved in DMSO at 10% and the initial concentration was 100 mg/ml. In each well, we poured 100 *µ*l of liquid culture medium (Mueller-Hinton), we added 100 *µ*l of extracts to be tested, and successive dilutions were then carried out. Each well is then inoculated with 10 *µ*l of the suspension of the microorganisms at final inoculum concentrations of 10^8^ CFU/ml. The plates were then covered with covers and incubated at 37°C for 24 hours.

After 24 hours of incubation, 10 *µ*l of MTT solution (3-(4,5-dimethylthiazol-2-yl)-2,5-diphenyl tetrazolium bromide) is added to each well, the plate is re-incubated for 15 minute at 37°C and the appearance of a violet stain shows bacterial growth. The MIC is the well concentration that exists just before the first purple-colored well [35].

#### 2.4.3. Determination of the Minimum Bactericidal Concentration (MBC)

To determine the MBC values, well solutions with an extract concentration equal to or higher than the MIC values were used. 10 *µ*l from each well was sub cultured on nutrient agar (Mueller-Hinton) in Petri dishes. The bacterial cultures were incubated at 37°C for 24 h. MBC was determined as the lowest concentration that showed no bacterial growth in the subcultures.

### 2.5. Statistical Analysis

The results were expressed as means ± standard error of triplicate determinations. Statistical analysis was performed by Graph Pad Prism by a one-way analysis of variance (ANOVA) followed by Tukey multiple comparison. A value of *p* < 0.05 was considered significant.

Correlation between the antioxidant activity and phytochemical composition of *Anabasis aretioïdes *Coss. & Moq. was carried out using Principal Component Analysis (PCA).

## 3. Results

### 3.1. Phytochemical Study of *Anabasis aretioïdes* Coss. & Moq

#### 3.1.1. The Yield of Aqueous and Organic Extracts

The aqueous extractions using distilled water, organic using Soxhlet with four solvents and cold maceration by methanol of the aerial part of *Anabasis aretioïdes* Coss. & Moq. allowed us to calculate the yield of each aqueous and organic extract. The results obtained are shown in Table 1.

Each extract is characterized by its color and its yield in relation to the dry matter and the extracts obtained have a pasty aspect, green or brown in color. The comparison of the yields of the different aqueous extracts shows that the best yield is obtained by maceration (3.41%), followed by decoction (3.12%) and then infusion (1.24%). For organic extracts, the best yields were obtained with the more polar solvent, methanol (3.39%), while the lowest yield was obtained with the least polar solvent, petroleum ether (0.43%).

#### 3.1.2. Phytochemical Screening

In order to search for the different classes of secondary metabolites in the powder and in aqueous and organic extracts of the aerial part from *Anabasis aretioïdes *Coss. & Moq., we carried out a phytochemical screening, by setting up a set of qualitative characterization reactions. These reactions are based on precipitation or coloration phenomena by specific reagents. The results of this phytochemical screening are reported in Table 2.

Phytochemical screening tests carried out on the powder of the aerial part of *Anabasis aretioïdes* Coss. & Moq. have revealed the presence of tannins (cathechic), saponins, and sterols in very high quantities. The three aqueous extracts are very rich in saponins and sterols, while the tannins (cathechic) are present but in small quantities. For organic extracts, phytochemical screening has revealed the presence of saponins and sterols in all extracts, the tannins (cathechic) are present in methanol extract, macerated methanol, and ethyl acetate extracts in high quantities; however, they are absent in the chloroformic and petroleum ether extracts. The search for flavonoids, alkaloids, anthracenosides, anthraquinones, and free quinones was revealed negative for the aqueous and organic extracts from aerial part of *Anabasis aretioïdes *Coss. & Moq. Similarly, these secondary metabolites are also absent in the powder thereof.

#### 3.1.3. Determination of Total Polyphenols and Cathechic Tannins

Quantitative analyses of total polyphenols and cathechic tannins are determined from the linear regression equations of each calibration curve expressed successively micrograms gallic acid equivalent and micrograms of catechin equivalent per mg of extract. The results of the colorimetric analysis of total phenolic compounds and total cathechic tannins are summarized in Table 3.

The results show that polyphenol contents vary considerably between aqueous and organic extracts of *Anabasis aretioïdes *Coss. & Moq. For the aqueous extracts, the decocted has the highest content with the value of 1.78 ± 0.003 *µ*g GAE/mg E, followed by the macerated 0.92 ± 0.03  *µ*g GAE/mg E, then the infused 0.65 ± 0.08 *µ*g GAE/mg E. For organic extracts, the results of the total phenolic contents show that levels vary between 46.79 ± 0.75 *µ*g GAE/mg E and 22.99 ± 0.19 *µ*g GAE/mg E. The largest amount of phenol was found in the ethyl acetate extract with the content of 46.79 ± 0.75 *µ*g GAE/mg E, followed by the chloroform extract 33.16 ± 0.53 *µ*g GAE/mg E, then 31.06 ± 0.19 *µ*g GAE/mg E for the methanol extract and 26.62 ± 0.17 *µ*g GAE/mg E for the methanol macerate, while petroleum ether extract contains only 22.99 ± 0.19 *µ*g GAE/mg E.

The determination of cathechin tannins showed that the highest content is that of ethyl acetate extract, which contains 46.46 ± 0.67 *µ*g CE/mg E, followed by petroleum ether extract 35.57 ± 1.95 *µ*g CE/mg E, chloroform extract 28.30 ± 1.40 *µ*g CE/mg E, macerated methanol extract 5.25 ± 0.58 *µ*g CE/mg E, and finally the methanol extract 4.10 ± 0.55 *µ*g CE/mg E, while aqueous extracts contain a low content of cathechic tannins which varies between 0.39 ± 0.02 *µ*g CE/mg E for macerated and 0.70 ± 0.03 *µ*g CE/mg E for infused, and 0.68 ± 0.09 *µ*g CE/mg E for decocted.

### 3.2. Biological Study of *Anabasis aretioïdes* Coss. & Moq.

#### 3.2.1. Determination of Antioxidant Activity

Antioxidant and antiradical activities were measured *in vitro* using five methods: H_2_O_2_, DPPH, ABTS, FRAP, and Reducing Power for each of our eight extracts and repeated three times to verify the reproducibility of the tests (Table 4).

*Hydrogen Peroxide Scavenging Activity. *We evaluated *Anabasis aretioïdes* Coss. & Moq. extracts on reactive oxygen species using hydrogen peroxide scavenging test. In Table 4, we noticed that all the aqueous and organic extracts of *Anabasis aretioïdes* showed a low hydrogen peroxide scavenging activity. Aqueous macerated extracts and methanolic extracts being the most active, with percentages of trapping in the order of 7.84 ± 0.44% and 5.32 ± 0.23%, respectively.

*DPPH Radical Scavenging Assay. *The IC_50_ values (the concentration of the sample tested required to reduce 50% of the DPPH radical) of the tested extracts and reference standards are shown in Table 4. These values allow us to evaluate and compare the efficacy of the different extracts of *Anabasis aretioïdes* Coss. & Moq. Indeed, the lower IC_50_ value, the higher is the antioxidant activity [36]. The highest DPPH scavenging activity of *Anabasis aretioïdes* Coss. & Moq. can be attributed to the methanol macerated extract with an IC_50_ of 52.91 ± 0.24 *µ*g/ml, followed by the methanol extract IC_50_=59.65 ± 1.67 *µ*g/ml, then the ethyl acetate extract with an IC_50_ of 76.08 ± 1.28 *µ*g/ml with a nonsignificant difference between the three extracts, then the petroleum ether extract IC_50_ = 515.53 ± 1.39 *µ*g/ml. On the other hand, the chloroformic extract has a low antiradical activity with an IC_50_ of 863.60 ± 10.49 *µ*g/ml.

The scavenging effect of aqueous extracts was very low compared to organic extracts; we found that the decocted presents the most important activity IC_50_ = 1117.67 ± 0.27 *µ*g/ml, followed by the macerated and infused extracts whose IC_50_ values are, respectively, in the order of 2704.33 ± 1.91 *µ*g/ml, 3704.33 ± 5.97 *µ*g/ml with a significant difference between the three extracts. On the other hand, statistical analysis revealed that there is a significant difference when comparing the three standard antioxidants and the aqueous and organic extracts of *Anabasis aretioïdes* Coss. & Moq. (*p* < 0.05). A comparison of the IC_50_s of our aqueous and organic extracts with the IC_50_s of standard antioxidants shows that all extracts have a low antioxidant capacity compared to the reference standards. Standard antioxidants have a high antioxidant capacity in the order of 0.17 ± 0.02 *µ*g/ml, 1.59 ± 0.13 *µ*g/ml, 1.75 ± 0.09 *µ*g/ml, respectively, for ascorbic acid, BHA, and Trolox.

*ABTS Radical Scavenging Assay. *In the present study, we evaluated the effect of aqueous and organic extracts of the aerial part from *Anabasis aretioïdes *Coss. & Moq. to scavenging ABTS radicals, from a calibration curve with R equal to 0.9968, we calculated the antiradical power of each extract which is expressed in μg equivalent of trolox per milligram of extract and the results are presented in Table 4.

For aqueous extracts, we distinguish that the best antiradical activity observed is that of macerated with a value of 1.56 ± 0.006 *µ*g TE/mg E, followed by decocted 1.45 ± 0.027 *µ*g TE/mg E, then infused (0.69 ± 0.093 *µ*g TE/mg E) with a nonsignificant difference between the three extracts.

A comparison of the activity of the organic extracts shows that macerated methanol extract is the most active (48.99 ± 1.316 *µ*g TE/mg E) and has almost similar activity to ethyl acetate extract (48.06 ± 0.93 *µ*g TE/mg E) with a nonsignificant difference, followed successively by the methanolic extract (39.10 ± 0.572 *µ*g TE/mg E) then other extracts, chloroformic (31.89 ± 1.17 *µ*g TE/mg E) and petroleum ether (10.61 ± 1.528 *µ*g TE/mg E).

*Ferric Reducing/Antioxidant Power FRAP Assay. *In the case of the FRAP assay, the antioxidant potentials obtained from the three aqueous extracts vary from 0.456 ± 0.045 *µ*g TE/mg E for the infused to 2.896 ± 0.209 *µ*g TE/mg E for the decocted with a nonsignificant difference between the three aqueous extracts. We have noticed that aqueous extracts have lower antioxidant potentials than organic extracts. For these, macerated methanol extract has a higher activity of 99.73 ± 3.57 *µ*g TE/mg E, followed by ethyl acetate extract 83.74 ± 6.34 *µ*g TE/mg E. Statistical analysis shows that these two extracts have antioxidant potentials with a nonsignificant difference, while chloroformic and petroleum ether extracts have a low activity with values of 50.19 ± 1.34 *µ*g TE/mg E, 24.60 ± 1.46 *µ*g TE/mg E respectively. For the extraction with the same solvent with methanol, we noted that the cold extraction (macerated methanol extract) gives the highest activity 99.73 ± 3.57 *µ*g TE/mg E compared to hot extraction 79.21 ± 2.03 *µ*g TE/mg E and compared to other organic extracts.

*Reducing Power Assay. * As shown in Table 4, the reducing power of all the organic extracts is stronger than in the aqueous extracts, we have also clearly observed that the classification of extracts according to their reducing capacity is similar to the classification obtained by the FRAP method. It is always the extract of methanolic macerated that has the highest reducing power with 72.176 ± 0.540 *µ*g AAE/mg E, followed by ethyl acetate extract (63.480 ± 3.701 * µ*g AAE/mg E) with a difference not significant. For the aqueous extracts, the values were 1.72 ± 0.04 *µ*g AAE/mg E, 0.53 ± 0.08 *µ*g AAE/mg E and 0.20 ± 0.03 *µ*g AAE/mg E for decocted, macerated, and infused, respectively.

#### 3.2.2. Antibacterial Activity of Organic Extracts from the Aerial Part of *Anabasis aretioïdes* Coss. & Moq. by the Disc Diffusion Test and Determination of MIC and MBC:

The study of the *in vitro* antibacterial activity of organic extracts from the aerial part of *Anabasis aretioïdes *Coss. & Moq. was carried out against 6 pathogenic microorganisms using the disc diffusion method. The results of the antibacterial activity of the organic extracts from aerial part of *Anabasis aretioïdes* Coss. & Moq.by the disc diffusion test are summarized in Table 5.

Table 5 shows the inhibition zone diameters of the different extracts tested on different bacterial strains, ranging from the lowest value of 7–13.5 mm. The results obtained show that the antibacterial activity of the extracts tested varies according to the targeted bacteria. The diameters of inhibition, obtained by organic extracts, are very variable depending on the extract used.

Methanolic and macerated methanol extracts showed inhibition of the growth of two tested microorganisms (2/6), *Proteus mirabilis* and *Bacillus subtilis* DSM6633. Indeed, the extract resulting from maceration and the extract obtained by Soxhlet showed moderate activities at a concentration of 100 mg/ml with an inhibition diameter of 9.5 mm and 11.5 mm, respectively, against *Protéus mirabilis* and low activities with an inhibition diameter of 8.5 mm and 7.5 mm, respectively against *Bacillus subtilis *DSM6633, while *Escherichia coli* K12, *Pseudomonas aeruginosa* CECT118, and *Staphylococcus aureus* CECT976 showed resistance to methanol macerate and methanol extract.

Ethyl acetate extract has antibacterial activity on 5/6 of the strains studied, its action is moderate against the strain *Staphylococcus aureus* CECT976, *Proteus mirabilis*, *Bacillus subtilis *DSM6633, *Escherichia coli* K12, and *Pseudomonas aeruginosa* CECT118, at the concentration of 100 mg/ml with inhibition diameters of 13.5 mm; 12.5 mm; 11.5 mm; 10.5 mm, and 8 mm, respectively. On the other hand, the chloroform extract has antibacterial activity on 5/6 of the bacteria studied and showed similar moderate activities with an inhibition diameter of 11.5 mm at the concentration 100 mg/ml against *Escherichia coli* K12, *Proteus mirabilis* and *Staphylococcus aureus* CECT976, it also inhibited the growth of *Bacillus subtilis* and *Pseudomonas aeruginosa* CECT118 with inhibition zone diameters of 10 mm and 8 mm, respectively, at the concentration of 100 mg/ml. Furthermore, it should be noted that at the concentration of 80, and 40 mg/ml, the inhibitory power of the active extracts has significantly decreased, which means that the antibacterial activity of the extracts studied is proportional to the concentration of bioactive substances they contain. The petroleum ether extract showed no activity against the 6 microorganisms tested at all concentrations of 100, 80, and 40 mg/ml and the *Listeria innocua* CECT 4030 strain was resistant to all organic extracts of *Anabasis aretioïdes *Coss. & Moq., this is related to their high capacity to develop resistance against many antimicrobial agents. All the antibacterial activities of the registered extracts remain lower than that of the reference antibiotic: tetracycline and amikacin.

The purpose of the micro-dilution method is to evaluate the minimum inhibitory concentrations and to determine the lowest concentration of an antibacterial agent necessary to inhibit the growth of a microorganism. The effectiveness of the extracts tested is evaluated by measuring two concentrations, the minimum inhibitory concentration (MIC) and the minimum bactericidal concentration (MBC). These concentrations allow us to know the nature of the antimicrobial activity: bacteriostatic or bactericidal.

The values of the minimum inhibitory concentration (MIC) and minimum bactericidal concentration (MBC) of the various extracts tested are grouped in Table 6.

According to Table 6, we observe that the cold methanolic macerated extract and the hot methanolic extract showed the lowest MIC value, which is in the order of 3.125 mg/ml for *Protéus mirabilis*, and the MIC value for *Bacillus subtilis* DSM 6633 in the order of 50 mg/ml. Ethyl acetate extract of *Anabasis aretioïdes *Coss. & Moq. has bacteriostatic activity against five bacterial strains (5/6), which had the lowest MICs. The strongest activity of ethyl acetate extract was demonstrated against *Proteus mirabilis* with a MIC equal to 6.25 mg/ml followed by a MIC value of 12.5 mg/ml for *Escherichia coli* K12, *Pseudomonas aeruginosa* CECT118, *Staphylococcus aureus* CECT976, and for *Bacillus subtilis *the MIC value is 25 mg/ml. On the other hand, the chloroformic extract has the same MIC for the strains *Pseudomonas aeruginosa*, *Staphylococcus aureus* CECT976, *Bacillus subtilis *DSM 6633 in the order of 100 mg/ml and 50 mg/ml *against Escherichia coli* K12 and *Proteus mirabilis*.

The evaluation of the MBC revealed that macerated methanol extract showed the highest bactericidal activity (MBC = 3.125 mg/ml) against *Proteus mirabilis* followed by methanol extract MBC = 6.25 mg/ml. For ethyl acetate extract, the MBCs on the strains tested are 25 mg/ml on *Staphylococcus aureus* CECT976 and *Bacillus subtilis *DSM 6633, 50 mg/ml for *Pseudomonas aeruginosa* CECT118 and 100 mg/ml for *Escherichia coli* K12. The chloroformic extract showed bactericidal activity (MBC = 100 mg/ml) against *Bacillus subtilis *DSM 6633 and *Escherichia coli* K12.

### 3.3. Principal Component Analysis (PCA)

In our study, Principal component analysis aims to establish the correlation between the different methods used to determine antioxidant activity on the one hand, and between the different families dosed in the plant on the other hand.

#### 3.3.1. Correlation Matrix

The Principal component analysis was carried out on a matrix that includes all the data from the different dosed families and the antioxidant activities by the five tests considered as variables (7 variables), individuals are represented by the 8 extracts (aqueous and organic) taking into account the different solvents and extraction methods used (Table 7).

#### 3.3.2. Characterization of Variables and Individuals

**Figure 1:**
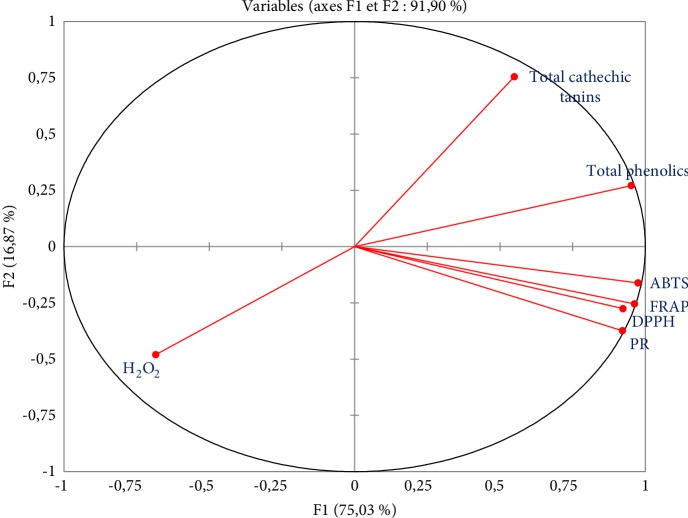
PCA factorial plan performed on the values of the different dosed families and antioxidant capacities estimated by five different methods.

On the F1-F2 factorial plan (Figure 1) are represented the projection of variables by the PCA concerning the different tests used and the results of phytochemical assays. The first main component (F1) explains 75.03% of the total information and the second main component (F2) shows 16.86%. The linear combination of the two first principal components is already representative of the variables because their cumulative percentage is 91.90% which is greater than 50%, which means that the first two axes are sufficient to represent the information as a whole. Figure 1 represents the plane formed by axes F1 and F2 giving the correlation between the variables.

The F1 axis is mainly constructed by the positive correlation between the ABTS, FRAP, DPPH, PR, H_2_O_2_ tests and the total polyphenol content. Axis F2 is formed by the cathechic tannins contents (Figure 1).

**Figure 2:**
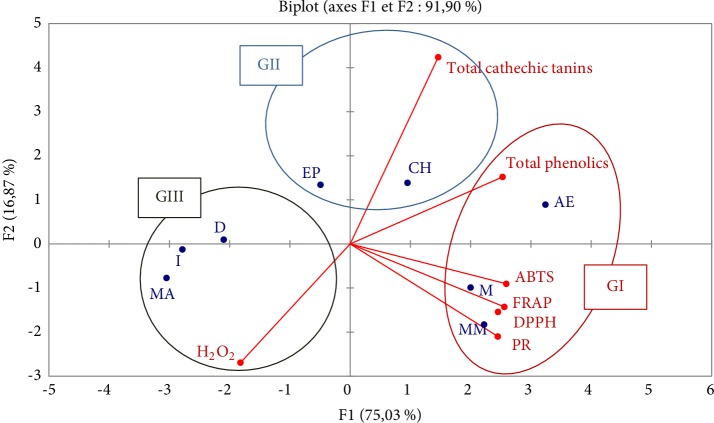
Projection of individuals on the factorial plan (F1 × F2). D: decocted; I: infused; MA: macerated; MM: methanol macerated extract; M: methanol extract; AE: ethyl acetate extract; CH: chloroform extract; EP: petroleum ether extract; GI: Group I; GII: Group II; GIII: Group III.

Figure 2 shows the distribution of the 8 individuals (extracts) into three groups.

This figure shows that the 8 individuals (8 extracts) of *Anabasis aretioïdes *Coss. & Moq. are divided into three groups:

1. Group I contains 3 extracts (methanol, macerated methanol and ethyl acetate), the antioxidant activities of the three extracts are higher than other aqueous and organic extracts by the four methods ABTS, DPPH, FRAP, and PR; the antioxidant capacity of these extracts is related to their total polyphenol content.
2. Group II is formed by 2 extracts (chloroform and petroleum ether); they are characterized by the high content of cathechic tannins and the antioxidant activity of these two extracts is low compared to Group I extracts.
3. Group III is made up of 3 individuals 3 aqueous extracts: decocted, infused, and macerated characterized by low polyphenol and tannin content, and therefore, low antioxidant activities.

## 4. Discussion

### 4.1. Phytochemical Study of *Anabasis aretioïdes* Coss. & Moq

#### 4.1.1. The Yield of Aqueous and Organic Extracts

The yields of aqueous extracts prepared from the aerial part of *Anabasis aretioïdes* Coss. & Moq. are 3.41%, 3.12%, and 1.24%, respectively, for maceration, decoction, and infusion. This result could be justified by the different conditions used in the three extraction methods, in particular temperature and extraction time. For decoction and infusion, it is necessary to work at high temperature to boil the water and the difference in yields can be explained by the temperature that remains high for decoction and gradually decreases for infusion. While the cold maceration process provides the best yield that can be explained by the extraction time that lasts 24 hours, while for decoction and infusion, the extraction time was only 20 minutes and 30 minutes, respectively. We can deduce that maceration can be used to extract the active ingredients from *Anabasis aretioïdes *Coss. & Moq. because it allows obtaining the best yield while preserving its constituents. Maceration with distilled water also allowed having the best yield compared to organic extracts.

The difference in the yield of organic extracts could be due to the extraction capacity of each solvent, each one of them can extract well-defined families of secondary metabolites existing in the aerial part of the plant studied. We will therefore note that the increase in the polarity of the solvent induces a significant improvement in the extraction efficiency yield. We have also noticed that there is a difference in yield between methanol and macerated methanol extract and that the hot extraction gives the best yield with organic solvents. This difference in yield can also be related to the extraction techniques used. We can therefore deduce that the more the solvent is polar the more it allows a better extraction and gives a better yield. Also, the temperature and the duration of extraction have an impact on the extraction efficiency.

The yields that we obtained are lower compared to those obtained from the roots of *Anabasis aretioïdes *from Algeria, which are in the order of 38.87% for the cold aqueous crude extract, 13.70% for the water/methanol crude extract, 5.096% for ethyl acetate and 2.395% for chloroformic extract [19]. Variations in yield can be attributed to the geographical origin of the plant, the part of the plant used, and to the extraction technique, but also to the period of harvest of the plant material and the extraction method [37, 38].

In addition, Benhammou and his collaborators evaluated the yield of extracts of *Anabasis articulata*, a plant of the same genus as *Anabasis aretioïdes*, harvested in the Bechar region (Algeria) and noted a yield of the methanolic extract of 5.47 ± 0.54% for the stem and 4 ± 0.04% for the root [39]. From these results, we can deduce that variations in extract yield could be attributed not only to the origin of the plant and the extraction technique but also to the species and part of the plant used.

#### 4.1.2. Phytochemical Screening

Phytochemical screening shows that *Anabasis aretioïdes *Coss. & Moq. harvested in Figuig city from eastern Morocco contains tannins (cathechic), saponins, and sterols. The results obtained by Bentabet and his collaborators reported the presence of alkaloids, tannins (condensed and hydrolyzable), reducing compounds and saponosides in the two parts (leaves and roots) of *Fredolia aretioïdes* from Algeria, while coumarins only appear in the leaves but in small quantities [19–40]. These differences in the chemical composition of *Anabasis aretioïdes* from Algeria and Morocco may be related to the method of preparation of the extracts, that means, the conditions under which the extraction is carried out (hot or cold) and the part of the plant used. In our study, we used the aerial part while in the Algerian study, they used leaves and roots. These differences can also be related to the harvesting season of the plant and especially to the geographical place of harvest, this is called chemotype. Indeed, we harvested our plant in September 2016 in the Figuig region (Morocco) while Bentabet harvested the plant in December 2011 in the Béchar region (Algeria). In addition, the use of extraction solvents with different polarities can affect the chemical composition of each extract, each solvent can separate molecules according to their degrees of solubility [37].

#### 4.1.3. Determination of Total Polyphenols and Cathechic Tannins

The comparison of the total polyphenol contents between the different extracts allowed us to establish this order: Ethyl acetate extract > Chloroform extract > Methanol extract > Methanol macerate extract > Petroleum ether extract > Decocted > Macerated > Infused.

For aqueous extracts, decoction seems to be the best method of hot and cold extraction of total polyphenols for aqueous extraction. Also the cold maceration has a higher content than the infused, so we can deduce the importance of the extraction time. When it is long, it can make it possible to extract the polyphenols better.

For organic extracts, the polyphenol content varies depending on the polarities of the solvent. According to our results, the use of ethyl acetate as an extraction solvent for *Anabasis aretioïdes *Coss. & Moq. polyphenols is recommended. In addition, since there is a significant difference between the polyphenol content in macerated methanol and methanol extract, we can say that the polyphenol content also varies according to the extraction method used cold or hot and it is better with Soxhlet extraction. Similarly, the decoction allowed to obtain the best polyphenol yield for aqueous extracts.

The work carried out by El-Haci and his collaborators on the aerial part of *Anabasis aretioïdes* harvested in November 2010 in Beni-Abbès (Béchar Regions, south-west Algeria) showed that the order of the total phenolic compound contents of hot organic extracts is: ethanol extract > Chloroform extract > Acetone extract > Ethyl acetate extract > Methanol extract, with a content of, respectively, 231.85 ± 20.59 mg GAE/g E, 196.63 ± 31.2 mg GAE/g E, 183.01 ± 4.38 mg GAE/g E, 134.82 ± 13.27 mg GAE/g E, and 101.85 ± 2.31 mg GAE/g E [18]. For the study conducted by Bentabet and his collaborators, the polyphenol content of water/methanol macerate (48 hours) of *Anabasis aretioïdes* is 764.54 ± 0.55 mg GAE/g at leaf level and 917.05 ± 0.83 mg GAE/g at the root level [40]. This suggests that the root is richer in polyphenols and that maceration is the best method of extraction and the extraction is better where the period of extraction is 48 hours.

Based on the results presented above and the literature, the polyphenol content in *Anabasis aretioïdes *may vary according to biotic conditions such as species, organ, physiological state, and abiotic conditions (season, climate, and temperature) [41]. It also depends on the method of hot or cold extraction by maceration and the extraction solvent used. For this purpose, the selection of the appropriate solvent remains one of the most important steps in the optimization of polyphenol extraction [42–37].

While for cathechic tannins, the extracts can be classified in this order: Ethyl acetate extract > Petroleum ether extract > Chloroform extract > Macerated methanol extract > Methanol extract > Infused >Decocted > Macerated. The extraction of tannins depends on the operating conditions, the extraction solvent used and the part of the plant [43]. A study conducted by Bentabet and his colleagues showed that the tannin content in hot acetone/water (70/30) hot extract of Algerian *Anabasis aretioïdes* is 29.97% in the leaves and stem level and 35.73% at the root level [44].

### 4.2. Biological Activities of *Anabasis aretioïdes* Coss. & Moq.

#### 4.2.1. Determination of Antioxidant Activity

*Hydrogen Peroxide Scavenging Activity. *Hydrogen peroxide is naturally formed as a by-product of oxygen-related metabolism in organisms. H_2_O_2_ is the most important source of ˙OH and causes lipid peroxidation due to the production of ˙OH. In the presence of Fe^+2^and other transition elements, the reactions of Fenton and Haber–Weiss cause the formation of the radical ˙OH, which is the most active and most harmful [45]. In this sense, the elimination of hydrogen peroxide is very important. We note that the various extracts have a low capacity to scavenging the H_2_O_2_ radical with percentages ranging from 2.81% to 7.84%. In addition, the aqueous and methanolic macerated extracts showed the most important antiradical activities, which confirms that maceration cold extraction makes it possible to better extract, or even preserve, the molecules responsible for the antioxidant activity of *Anabasis aretioïdes *Coss. & Moq. The work done by El-Haci and his collaborators showed that the methanolic, chloroformic, and ethyl acetate extract of the leaves of *Anabasis aretioïdes* of Algeria have an important capacity to eliminate radical hydrogen peroxide with a percentage of 26.98 ± 2.99, 29.28 ± 5.04, and 45.49 ± 3.84%, respectively [18].

*DPPH Radical Scavenging Assay. *DPPH is generally one of the most widely used substrate for the rapid and direct evaluation assessment of antioxidant activity due to its stability in radical form and the possibility of its analysis [46].

Depending on the results recorded, aqueous and organic extracts have a different activity to give the proton to neutralize the DPPH radical which decreases in the following manner: Methanol macerated extract > Methanol extract > Ethyl acetate extract > Petroleum ether extract > Chloroform extract > Decocted > Infused > Macerated.

The study conducted by El-Haci and his collaborators, on Algerian *Anabasis aretioïdes* showed by the same test as the ethyl acetate, methanolic and chloroformic extracts of the aerial part of *Anabasis aretioïdes* of Algeria have an important power free radical scavenger DPPH with IC_50_ equal to 72.15 ± 1.04 *µ*g/ml, 79.15 ± 4.23 *µ*g/ml and 86.73 ± 10.68 *µ*g/ml respectively. We have noticed that the IC_50_ values of the ethyl acetate and methanolic extracts of Algerian *Anabasis aretioïdes* and Moroccan *Anabasis aretioïdes* are almost similar, but there is a big difference between the IC_50_ values of chloroformic extracts, the IC_50_ is 863.60 ± 10.49 *µ*g/ml for Moroccan *Anabasis aretioïdes* and 86.73 *µ*g/ml for that of Algeria [18].

The work done by Bentabet and his collaborators showed that the aqueous extract of the roots of *Anabasis aretioïdes* from Algeria has an activity to trap the DPPH radical with an IC_50_ of 1810 ± 0.841 *µ*g/ml that can be explained by the high polyphenol content of the root [19].

A comparison of our results on *Anabasis aretioïdes* with another *Anabasis* species from Iran: *Anabasis aphylla* showed that the aerial part of this plant has no antioxidant activity by the DPPH test [47].

Benhammou and his collaborators reported that the methanolic extract of *Anabasis articulata*, harvested in Algeria, had a better scavenging efficiency against DPPH radicals with an IC_50_ of 570 ± 0.03 *µ*g/ml for root methanolic extract and 1980 ± 0.15 *µ*g/ml for stem methanolic extract [39].

Our results revealed that *Anabasis aretioïdes* extracts have a good ability to eliminate free radicals and prevent lipid peroxidation, which can be attributed to the high content of phenolic compounds in this plant as revealed by the phytochemical study. In fact, various studies have shown the correlation between phenolic content and antioxidant capacity of plant extracts [48, 49].

*ABTS Radical Scavenging Assay. *Similar to the DPPH test, the ABTS test is another widely used *in vitro* radical scavenging test. However, this method requires the generation of ABTS radicals which can be easily achieved by reacting ABTS salt with potassium persulfate. The ABTS radical cation is reactive towards most antioxidant compounds. The ABTS radical is soluble in aqueous and organic solvents. The method is useful for determining the antioxidant potential of lipophilic and hydrophilic antioxidants in various samples, such as plant extracts. A compound having the property of giving electrons will reduce the ABTS blue-green radical solution to a colorless neutral form [50].

The classification of the different extracts according to their antioxidant activity according to the ABTS test in descending order is as follows: Macerated methanol > Ethyl acetate extract > methanol extract > Chloroformic extract > Petroleum ether extract > Macerated > Decocted > Infused. From the results obtained, we noticed that the three polar extracts have a high capacity to scavenge the ABTS radical as in the DPPH test. This is due to the similarity of the two methods that measure the ability of antioxidants to give hydrogen or electron atom [51], but the ABTS method is more reliable than the DPPH method due to the solubility of the ABTS reagent in both aqueous and organic solvents compared to the DPPH [52]. For this reason, the ABTS test is better than the DPPH test when applied to a variety of plant foods containing hydrophilic, lipophilic molecules, and highly pigmented antioxidant compounds [53]. The results obtained allow us to deduce that the anti-radical activity increases with the polarity of the solvent used. Aqueous extracts have a lower antioxidant activity than organic extracts; such a variation shows the impact of extraction methods on the extraction of antioxidant compounds. This can be explained by the difference in experimental conditions in the extraction methods, in particular the nature of the solvent, the temperature, and the extraction time.

We also noted that the two aqueous and methanolic macerate extracts are the most active compared to the other extracts, these data indicate that the cold maceration process always gives extracts rich in antioxidant molecules. Indeed, temperature can cause thermal degradation of antioxidants, and are preserved during cold extraction by maceration.

Tahar and his collaborators studied the antioxidant activity of polyphenolic extracts from two plants: *Atriplex halimus* L. and *Haloxylon scoparium *pomel (family *Chenopodiaceae*) growing in Algeria. The butanol fraction of these two plants has a higher activity to scavenge the cation radical ABTS with an IC_50_ in the order of 0.202 mg/ml for the butanol fraction of *Atriplex halimus* L. and 0.003 mg/ml for the butanol fraction of *Haloxylon scoparium* pomel [54].

*Ferric Reducing/Antioxidant Power FRAP Assay. *The antioxidant potentials of aqueous and organic extracts of the aerial part of *Anabasis aretioïdes *Coss. & Moq. have been estimated based on their ability to reduce the TPTZ-Fe (III) complex to TPTZ-Fe (II).The ability of a compound to transfer electrons is a significant indicator of its potential as an antioxidant. This indicates that the antioxidant compounds of *Anabasis* are electron donors and could reduce the oxidized intermediate of lipid peroxidation processes; thus acting as primary and secondary antioxidants. This indicates that the antioxidant compounds of *Anabasis aretioïdes *Coss. & Moq. are electron donors and could reduce the oxidized intermediate of lipid peroxidation processes; acting as primary and secondary antioxidants [32]. The results of the FRAP test are similar to those found in the DPPH and ABTS tests, where the cold maceration method was more effective for the extraction of antioxidant compounds.

*Reducing Power Assay. *Several dosages are designed and used to determine overall antioxidant activity as an indication of the total capacity to resist the negative effect of stress induced by free radical formation. The reducing potential reflects the electron donor capacity associated with antioxidant activity. The presence of reducers (antioxidants) in the samples results in the reduction of the ferric complex to iron form and this reductive potential of the sample can be determined by the direct reduction of Fe [(CN) 6] 3 to Fe [(CN) 6] 2. The addition of Fe3+ free to the reduced product results in the formation of the complex, Fe4 [Fe (CN) 6] 3, which has a high absorbency at 700 nm [55]. The reduction of Fe (III) is often used as an indicator of electron donor activity.

According to this test, macerated methanol has the highest reducing power. These results prove that the extract of methanolic macerate is rich in reducing compounds, such as phenolic compounds that are responsible for total antioxidant activity. The reducing power is a very important aspect for the estimation of antioxidant activity [56].

Bentabet and his collaborators showed that the aqueous extract and alkaloid extract of the roots of *Anabasis aretioïdes* from Algeria have a good reducing capacity to iron by the reducing power test with optical density (OD) values of 0.891, and 0.892, respectively [19]. The species *Anabasis articulata* harvested in Algeria was also the subject of a single study on the reducing power of iron in methanolic extract using only the RP test with an IC_50_ of 0.66 ± 0.00 and 0.36 ± 0.00 mg/ml, respectively, for stem and root [39].

#### 4.2.2. Antibacterial Activity of Organic Extracts from the Aerial Part of *Anabasis aretioïdes* Coss. & Moq. by the Disc Diffusion Test and Determination of MIC and MBC

Antibacterial tests showed variable effects of the extracts tested against bacterial strains and inhibition zones ranged from 7 to 13.5 mm. Extracts are considered active if they produce microbial growth inhibition diameters greater than or equal to 15 mm and with inhibition diameters less than 15 mm, extracts have intermediate activity on the bacteria tested [57]. Ethyl acetate extract has the highest inhibition zone against *Staphylococcus aureus* CECT976 strain, which is positively correlation with their total phenol contents 46.79 ± 0.75 *µ*g GAE/mg E (Table 3). In addition, our results consolidated those reported in the literature that phenolic compounds show the greatest antibacterial activity and that the *Staphylococcus aureus* strain is particularly sensitive to phenolic compounds [58].

In the calculated MBC/MIC ratio, the extract is validated as bactericidal when it is less than or equal to 4 and bacteriostatic when it is greater than 4 [59]. Values in this ratio are variable for the various extracts according to the bacterial strains tested (Table 6), which makes it possible to classify these extracts according to their spectrum of action. Cold methanolic macerated extract and hot methanolic extract are bactericidal on *Proteus mirabilis* and *Bacillus subtilis *DSM6633. Ethyl acetate extract is bactericidal against three strains: *Pseudomonas aeruginosa* CECT118, *Staphylococcus aureus* CECT976, *Bacillus subtilis *DSM6633 and bacteriostatic against *Escherichia coli *K12. On the other hand, chloroformic extract has a bactericidal power on *Escherichia coli* K12 and *Bacillus subtilis *DSM6633.

The literature refers to two studies on the antibacterial potential of different extracts of the aerial part of Algerian *Anabasis aretioïdes*:

El-Haci in 2014 investigated the antimicrobial potential of five extracts (methanolic, ethanolic, acetone, ethyl acetate, chloroformic) of the aerial part of *Anabasis aretioïdes* from Algeria on Gram-positive and Gram-negative bacteria and noted that all organic extracts of the aerial part of *Anabasis aretioïdes* did not have any antimicrobial effect on the microbial strains tested [60].

Bentabet and his collaborators in 2014 showed that the hydromethanolic extract of the aerial part of *Anabasis aretioïdes* from Algeria shows a good activity against *Pseudomonas aeruginosa*, *Klebsiella pneumoniae* and *Acinobacter baumanii* with zones of inhibitions of 14 and 13 mm, respectively, and *Pseudomonas aeruginosa* was very sensitive to the ethyl acetate fraction, the inhibition diameter of 20 mm. On the other hand, these authors found that extracts of the aerial part of *Fredolia aretioides* are more active on Gram negative bacteria than on Gram-positive bacteria. MICs range from 0.68 to 3.75 mg/ml in Gram-negative bacteria and from 1.25 to 2.75 mg/ml in Gram-positive bacteria. The water/methanol and ethyl acetate extracts showed the best MICs against *Pseudomonas aeruginosa* with 0.87 and 0.68 mg/ml, respectively [44].

### 4.3. Principal Component Analysis (PCA)

Table 7 shows the Pearson correlation that allowed for a better understanding and analysis of the potential relationships between the different variables analyzed. It appears from this that the four methods (ABTS, DPPH, FRAP, and PR) used to determine the antioxidant capacities are highly correlated. We observed a positive correlation between ABTS and FRAP (*r*^2^ = 0.9891), FRAP and PR (*r*^2^ = 0.9796), ABTS and PR (*r*^2^ = 0.9632), DPPH and FRAP (*r*^2^ = 0.9440), DPPH and PR (*r*^2^ = 0.9439) and between DPPH and ABTS (*r*^2^ = 0.9050). This strong correlation indicates that in an extract, the bioactive molecules are providing the scavenging power of free radicals (DPPH and ABTS) are themselves responsible for the reducing power of iron (FRAP and PR). Thus in our extracts, antioxidant molecules that can be involved through two types of reaction mechanisms. It should be noted that the reactions involved may differ from one test to another.

For the FRAP and PR tests, it is a reduction of Fe (III), therefore, based exclusively on electron transfer. Concerning the DPPH and ABTS tests, these two radicals can be neutralized either by direct reduction by electron transfers or by radical scanning by a transfer of a hydrogen atom [51]. Negative correlations were observed between the H_2_O_2_ test and the 4 tests (ABTS, DPPH, FRAP, PR), which is in agreement with the results of the experimental part, in fact the different extracts have a low capacity to scavenge the H_2_O_2_ radical with very low percentages of inhibitions.

We also noted a positive correlation between polyphenol content and ABTS (*r*^2^ = 0.8914), FRAP (*r*^2^ = 0.8502), DPPH (*r*^2^ = 0.7916) and PR (*r*^2^ = 0.7766) tests. The antioxidant activity of the extracts tested is related to the content of phenolic compounds present in the plant. These results are in agreement with what has been reported in the literature by several authors that the antioxidant activity potential of an extract depends on its content of phenolic compounds [61–62].

A positive correlation also observed between the cathechic tannin content and the polyphenol content (*r*^2^ = 0.7514) shows that cathechic tannins, the most important group of phenols in the *Anabasis aretioïdes* plant. On the other hand, the tannin content is very poorly correlated with antioxidant activity, so it is clear that the antioxidant activity of our extracts is due to the total polyphenol content they contain and not to their cathechic tannin content. Negative correlations were also observed between the H_2_O_2_ test and the two dosed families, we can deduce that there are other families of secondary metabolites in extracts capable of eliminating hydrogen peroxide.

Figure 2 shows that the 8 individuals (extracts) are divided into three groups:

Group I consists of 3 extracts (methanol, macerated methanol, and ethyl acetate), the antioxidant activities of the three extracts is significantly higher than other aqueous and organic extracts by the four methods ABTS, DPPH, FRAP, and PR. The antioxidant capacities of the extracts are proportional to the polarity of the solvents used. Similarly, Kang and his team (2003) suggested that plant extracts containing polar molecules show high antiradical activities [63]. The extracts resulting from maceration have a higher antioxidant activity than the extracts obtained by Soxhlet. Maceration makes it possible to better extract the molecules responsible for antioxidant activity. The cold maceration process was carried out at ambient temperature, whereas in the Soxhlet process, it is necessary to work at high temperature to boil the solvent, which would result to the thermal degradation of thermosensitive compounds [64].

Group II is formed by 2 extracts (chloroform and petroleum ether), characterized by the high content of cathechic tannins. Therefore, the extraction solvent system would influence the contents of cathechic tannins, chloroform extracts tannins better. This would be explained by the fact that cathechic tannin extraction yields are higher with low-polarity solvents, and this would be explained by the fact that the extraction yields of cathechic tannins are higher with solvents of which the polarity is lower than that of water.

Group III is made up of 3 individuals, three aqueous extracts (decocted, infused, macerated) characterized by the low content of polyphenols and tannins, which shows that aqueous extraction by three modalities (decoction, infusion, maceration) allows to extract less phenolic compounds responsible for antioxidant activities. All aqueous and organic extracts are not very active against hydrogen peroxide; aqueous macerate extract is the most active by H_2_O_2_ test.

## 5. Conclusion

The present work is devoted to the determination of the yield, chemical composition, antioxidant and antiradical properties, and antibacterial effect of *Anabasis aretioïdes *Coss. & Moq. extracts from eastern Morocco. The highest yield is obtained by aqueous macerated extract (3.41%) and by methanolic extract for the organic extracts (3.39%). Phytochemical screening revealed the richness of our plant in saponins, cathechic tannins, and sterols which are present in large quantities in the powder of the plant material and in all extracts prepared. In addition, the quantitative analysis revealed significant levels of total polyphenols and cathechic tannins, particularly in ethyl acetate extract.

Evaluation of the antioxidant activities of aqueous and organic extracts of *Anabasis aretioïdes* Coss. & Moq. has made it possible to select methanolic macerated as the extract with the best antioxidant activities. The principal component analysis indicates the existence of a strongly positive correlation on the one hand, between the four methods ABTS, DPPH, FRAP, and PR, and on the other hand, between the antioxidant capacities of the extracts and the total polyphenol content.

Antibacterial tests showed that ethyl acetate extract showed the highest inhibition against *Staphylococcus aureus* strain with an inhibition diameter of 13.5 mm. The methanolic and macerated methanolic extracts gave the same MIC value (3.125 mg/ml) for *Proteus mirabilis* strain, which is the lowest MIC.

The results obtained are very encouraging and promising both in terms of anti-radical and antibacterial activities, particularly for methanolic macerated extract. For this extract, we can consider the *in vivo* evaluation of antioxidant activities.

## Figures and Tables

**Table 1:** Yields of aqueous and organic extractions of aerial part of *Anabasis aretioïdes*.

Extract		Yield (%)
Aqueous	Decocted	**3.12**
	Infused	1.24
	Macerated	**3.41**
		
Organic	Methanol extract	**3.39**
	Macerated methanol	2.58
	Ethyl acetate extract	2.45
	Chloroform extract	1.14
	Petroleum ether extract	0.43

**Table 2:** Phytochemical screening performed on powder, aqueous, and organic extracts of aerial part of *Anabasis aretioïdes*.

Plant and extract	Cathechic tannins	Gallic tannins	Flavonoids	Saponines	Alkaloids	Sterols	Anthaceno-sides	Anthraquinone	Free quinones
*Powder from vegetable material*	+++	−	−	+++	−	+++	−	−	−
*Decocted*	+	−	−	+++	−	+++	−	−	−
*Infused*	+	−	−	+++	−	+++	−	−	−
*Macerated*	+	−	−	+++	−	+++	−	−	−
*Methanol*	+++	−	−	+++	−	+++	−	−	−
*Macerated methanol*	+++	−	−	+++	−	+++	−	−	−
*Ethyl acetate*	+++	−	−	++	−	+++	−	−	−
*Chloroform*	−	−	−	+++	−	+++	−	−	−
*Petroleum ether*	−	−	−	+++	−	+++	−	−	−

+++: very abundant ++: moderately abundant +: present −: absent

**Table 3:** Polyphenol and cathechic tannin content of aqueous and organic extracts of aerial part of *Anabasis aretioïdes*.

Extract	Aqueous			Organic				
	Decocted	Infused	Macerated	Methanol	Macerated methanol	Ethyl acetate	Chloroform	Petroleum ether
*Total phenolics* (*µ*g GAE/mg E)	**1.78 ± 0.003** ^a^	0.65 ± 0.08^a^	0.92 ± 0.03^a^	31.06 ± 0.19^b^	26.62 ± 0.17^c^	**46.79 ± 0.75** ^d^	33.16 ± 0.53^e^	22.99 ± 0.19^f^
*Cathechic tannins content* (*µ*g CE)/mg E)	0.68 ± 0.09^a^	**0.70 ± 0.03** ^a^	0.39 ± 0.02^a^	4.10 ± 0.55^a^	5.25 ± 0.58^a^	**46.46 ± 0.67^c^**	28.30 ± 1.40^d^	35.57 ± 1.95^e^

All results expressed are mean of three individual replicates (*n* = 3 ± SEM). Values with the same letters superscript in the same row are not significantly different (*p* < 0.05).

**Table 4:** Antioxidant and antiradical activities of aqueous and organic extracts of aerial part of *Anabasis aretioïdes*.

Extracts	H_2_O_2 _scavenging activity (%)	DPPH IC_50_ (*µ*g/ml)	ABTS (*µ*g TE/mg E)	FRAP (*µ*g TE/mg E)	RP (µg AAE/mg E)
Decocted	4.52 ± 0.69^a,d^	1117.67 ± 0.27^a^	1.45 ± 0.027^a^	2.896 ± 0.209^a^	1.727 ± 0.047^a^
Infused	**5.96 ± 0.19** ^b,d^	3704.33 ± 5.97^b^	0.69 ± 0.093^a^	0.456 ± 0.045^a^	0.204 ± 0.031^a^
Macerated	**7.84 ± 0.44** ^c^	2704.33 ± 1.91^c^	1.56 ± 0.006^a^	1.790 ± 0.008^a^	0.539 ± 0.081^a^
Macerated methanol	**5.32 ± 0.23** ^d^	**52.91 ± 0.24** ^d^	**48.99 ± 1.316** ^b^	**99.736 ± 3.570** ^b^	**72.176 ± 0.540** ^b^
Methanol	3.81 ± 0.26^d^	**59.65 ± 1.67** ^d^	**39.10 ± 0.572** ^c^	**79.214 ± 2.031** ^c^	**59.954 ± 1.505** ^c^
Ethyl acetate	3.65 ± 0.80^d^	**76.08 ± 1.28** ^d^	**48.06 ± 0.93** ^b^	**83.743 ± 6.346** ^d,b^	**63.480 ± 3.701** ^d,b^
Chloroform	2.81 ± 0.43^e,d^	863.60 ± 10.49^e^	31.89 ± 1.17^d^	50.199 ± 1.341^e^	23.376 ± 1.601^e^
Petroleum ether	**4.91 ± 0.38** ^d^	515.53 ± 1.39^f^	10.61 ± 1.528^e^	24.601 ± 1.466^f^	4.640 ± 0.099^a^
Ascorbic acid	14.35 ± 0.002^f^	0.17 ± 0.02^g^	−	−	−
BHT	−	1.59 ± 0.13^g^	−	−	−
Trolox	−	1.75 ± 0.09^g^	−	−	−

All results expressed are mean of three individual replicates (*n* = 3 ± SEM). Values with the same letters superscript in the same column are not significantly different (*p* < 0.05).

**Table 5:** Antibacterial activity of organic extracts from the aerial part of *Anabasis aretioïdes*.

Inhibition zone (mm)																	
Extract Strains	Macerated methanol			Methanol			Ethyl acetate			Chloroform			Petroleum ether			Control (+)	Control (−)
	40 mg/ml	80 mg/ml	100 mg/ml	40 mg/ml	80 mg/ml	100 mg/ml	40 mg/ml	80 mg/ml	100 mg/ml	40 mg/ml	80 mg/ml	100 mg/ml	40 mg/ml	80 mg/ml	100 mg/ml	T/AK 20/30 *µ*g/ml	DMSO (10%)

*E. coli*	−	−	−	−	−	−	−	9	10.5	−	−	11.5	−	−	−	16/T	−
Pm	7.5	7.5	9.5	7	7.5	**11.5**	8.5	8	**12.5**	8	8	**11.5**	−	−	−	25.5/T	−
Pa	−	−	−	−	−	−	7	−	8	7	7	8	−	−	−	23/AK	−
Sa	−	−	−	−	−	−	10	10	**13.5**	7	8	**11.5**	−	−	−	14/T	−
Bs	−	7	8.5	−	7	7.5	8	**9**	**11.5**	7	9.5	10	−	−	−	10.5/T	−
Lis	−	−	−	−	−	−	−	−	−	−		−	−	−	−	−	−

*E. coli*: *Escherichia coli* K12; Pm: *Proteus mirabilis*; Pa: *Pseudomonas aeruginosa* CECT 118; Sa: *Staphylococcus aureus* CECT 976; Bs: *Bacillus subtilis* DSM 6633, Lis: *Listeria innocua* CECT 4030, (−): absence of inhibition; AK: amikacin; T: tétracycline; DMSO: dimethylsulfoxide.

**Table 6:** MIC and MBC (mg/ml) of organic extracts from aerial part of *Anabasis aretioïdes*.

Extract	Macerated methanol			Methanol			Ethyl acetate			Chloroform		
Strains	MIC mg/ml	MBC mg/ml	MBC/MIC	MIC mg/ml	MBC mg/ml	MBC/MIC	MIC mg/ml	MBC mg/ml	MBC/MIC	MIC mg/ml	MBC mg/ml	MBC/MIC
*E. coli*	-	-	−	−	−	−	12.5	100	8	50	100	2
Pm	**3.125**	**3.125**	1	**3.125**	**6.25**	2	**6.25**	ND	−	50	ND	−
Pa	−	−	−	−	−	−	12.5	50	4	100	ND	−
Sa	−	−	−	−	−	−	12.5	25	2	100	ND	−
Bs	50	50	1	50	50	1	25	25	1	100	100	1

*E.coli: Escherichia coli* K12; Pm: *Proteus mirabilis;* Pa: *Pseudomonas aeruginosa* CECT 118; Sa: *Staphylococcus aureus* CECT 976; Bs: *Bacillus subtilis* DSM 6633; MIC: minimum inhibitory concentration; MBC: minimum bactericidal concentration; MBC/MIC ≤4: bactericidal power; MBC/MIC >4: bacteriostatic power.

**Table 7:** Correlation matrix between phytochemical data and antioxidant activities of *Anabasis aretioïdes*.

Variables	Total phenolics	Cathechic tanins	H_2_O_2_	ABTS	FRAP	PR	DPPH
Total phenolics	**1**						
Cathechic tanins	**0.7514**	**1**					
H_2_O_2_	**−0.7295**	**−**0.5471	**1**				
ABTS	**0.8914**	0.4167	**−**0.5751	**1**	**0.9891**	**0.9632**	**0.9050**
FRAP	**0.8502**	0.3447	**−**0.5194	**0.9891**	**1**	**0.9796**	**0.9440**
PR	**0.7766**	0.2350	**−**0.4357	**0.9632**	**0.9796**	**1**	**0.9439**
DPPH	**0.7916**	0.3159	**−**0.4930	**0.9050**	**0.9440**	**0.9439**	**1**

The values in bold are different from 0 at a significance level alpha = 0.05.

## Data Availability

All data and materials supporting the conclusion in this paper are described and included in this published article.

## References

[1] Tenover F. C. (2006). Mechanisms of antimicrobial resistance in bacteria. *The American Journal of Medicine*.

[2] Percival M. (1997). Phytonutrients and detoxification. *Clinical Nutrition Insights*.

[3] Aruoma O. I. (1998). Free radicals, oxidative stress, and antioxidants in human health and disease. *Journal of the American Oil Chemists’ Society*.

[4] Michael K., Toby L., Nizet V. (2006). Innate immunity gone awry: linking microbial infections to chronic inflammation and cancer. *Cell*.

[5] Berrino F., Verdecchia A., Lutz J. M., Lombardo C., Micheli A., Capocaccia R. (2009). Comparative cancer survival information in Europe. *European Journal of Cancer*.

[6] Forni C., Facchiano F., Bartoli M. (2019). Beneficial role of phytochemicals on oxidative stress and age-related diseases. *BioMed Research International*.

[7] Middleton E., Kandaswami C., Theoharides T. C. (2000). The effects of plant flavonoids on mammalian cells: implications for inflammation, heart disease and cancer. *Pharmacological Reviews*.

[8] Ksouri R., Megdiche W., Debez A., Falleh H., Grignon C., Abdelly C. (2007). Salinity effects on polyphenol content and antioxidant activities in leaves of the halophyte *Cakile maritima*. *Plant Physiology and Biochemistry*.

[9] Nijveldt R. J., Van Nood E., Van Hoorn D. E., Boelens P. G., Van Norren K., Van Leeuwen P. A. (2001). Flavonoids: a review of probable mechanisms of action and potential applications. *The American Journal of Clinical Nutrition*.

[10] Haddouchi F., Chaouche T. M., Zaouali Y., Ksouri R., Attou A., Benmansour A. (2013). Chemical composition and antimicrobial activity of the essential oils from four *Ruta species* growing in Algeria. *Food Chemistry*.

[11] Benayad N., Mennane Z., Charof R., Hakiki A., Mosaddak M. (2013). Antibacterial activity of essential oil and some extracts of *Cistus ladaniferus* from Oulmes in Morocco. *Journal of Materials and Environmental Science*.

[12] Fennane M. (2004). Important plant areas in Morocco, proposals for important plant areas in Morocco. https://www.yumpu.com/fr/document/read/33732868/propositions-de-zones-importantes-pour-les-plantes-au-maroc.

[13] Ben El Mostafa S., Haloui B., Berrichi A. (2001). Endemic Moroccan and Moroccan-Algerian plants present in the Horsts and Monts de Debdou chain (oriental Morocco). *Bulletin mensuel de la Société linnéenne de Lyon*.

[14] Kaabeeche M. (2006). Fredolia aretioides, botanical curiosity of the taghit park, biodiversity conservation and sustainable management of natural resources (living nature).

[15] Bnouham M., Mekhfi H., Legssyer A., Ziyyat A. (2002). Medicinal plants used in the treatment of diabetes in Morocco. *International Journal of Diabetes and Metabolism*.

[16] El Mansouri L., Ennabili A., Bousta D. (2011). Socioeconomic interest and valorization of medicinal plants from the Rissani oasis (SE of Morocco). *Boletin Latinoamericano Caribe de Plantas Medicinales Aromaticas*.

[17] Lamchouri F., Zahidy M., Settaf A., Cherrah Y., Slaoui M., Hassar M. (1999). Comparative study of the antimitotic activity of the decoction of *Anabasis aretioides*, Haloxylon scoparium and Peganum harmala. *Espérance Médicale*.

[18] El-Haci I. A., Bekkara F. A., Mazari W., Gherib M. (2013). Phenolics content and antioxidant activity of some organic extracts of endemic medicinal plant *Anabasis aretioides* Coss & Moq. from Algerian Sahara. *Pharmacognosy Journal*.

[19] Bentabet N., Boucherit-Otmani Z., Boucherit K. (2014). Chemical composition and antioxidant activity of organic extracts of the roots of Fredolia aretioides from the Béchar region of Algeria. *Phytothérapie*.

[20] N’Guessan K., Kadja B., Zirihi G., Traoré D., Aké-Assi L. (2009). Screening phytochimique de quelques plantes médicinales ivoiriennes utilisées en pays Krobou (Agboville, Côte-d’Ivoire). *Sciences & Nature*.

[21] Karumi Y., Onyeyili P. A., Ogugbuaja V. O. (2004). Identification of active principales of M Balsamina (Balsam Apple) leaf extract. *Journal of Medical Sciences*.

[22] Bekro Y.-A., Mamyrbekova J. A., Boua B. B., Tra Bi F. H., Ehile E. E. (2008;). Ethnobotanical study and phytochemical screening of Caesalpinia benthamiana (Baill.) Herend. and Zarucchi (Caesalpiniaceae). *Sciences & Nature*.

[23] Reifer L., Niziolek S. (1957). Colorimetric microdetermination of alkaloids in lupine seeds. *Acta Biochimica Polonica*.

[24] Houta O., Chouaeb H., Neffati M., Amri H. (2012). Criblage chimique preliminaire des proteines et carotenoides presents dans un *Crithmum maritimum* cultive en tunisie. *Journal de la Société Chimique de Tunisie*.

[25] Suleiman M. H. A., Ahmed Y. A. I., Osman A. A. (2016). Screening of anthraquinones and assessment of antimicrobial activities of ethanol extracts of *Adansonia digitata* L., Sudan. *International Journal of Science and Research*.

[26] Dohou N., Yamni K., Tahrouch S., Idrissi Hassani L. M., Badoc A., Gmira N. (2003). Phytochemical screening of an Ibero-Moroccan endemic, thymelaea lythroides. *Bulletin de la Société de pharmacie de Bordeaux*.

[27] Lister E., Wilson P. (2001). *Measurement of Total Phenolics and ABTS Assay for Antioxidant Activity (Personal Communication)*.

[28] Julkunen-Tiitto R. (1985). Phenolic constituents in the leaves of northern willows: methods for the analysis of certain phenolics. *Journal of Agricultural and Food Chemistry*.

[29] Ruch R. J., Cheng S., Klaunig J. E. (1989). Prevention of cytotoxicity and inhibition of intercellular communication by antioxidant catechins isolated from Chinese green tea. *Carcinogenesis*.

[30] Sharma O. P., Bhat T. K. (2009). DPPH antioxidant assay revisited. *Food Chemistry*.

[31] Re R., Pellegrini N., Proteggente A., Pannala A., Yang M., Rice-Evans C. (1999). Antioxidant activity applying an improved ABTS radical cation decolorization assay. *Free Radical Biology and Medicine*.

[32] Benzie I. F. F., Strain J. J. (1996). The ferric reducing ability of plasma (FRAP) as a measure of “Antioxidant Power”: the FRAP assay. *Analytical Biochemistry*.

[33] Oyaizu M. (1986). Studies on products of browning reaction prepared from glucoseamine. *Japanese Journal of Nutrition and Dietetics*.

[34] Sharififar F., Moshafi M. H., Mansouri S. H., Khodashenas M., Khoshnoodi M. (2007). *In vitro* evaluation of antibacterial and antioxidant activities of the essential oil and methanol extract of endemic *Zataria multiflora* Boiss. *Food Control*.

[35] Gulluce M., Sahin F., Sokmen M. (2007). Antimicrobial and antioxidant properties of the essential oils and methanol extract from *Mentha longifolia* L. ssp. *longifolia*. *Food Chemistry*.

[36] Khoudali S., Benmessaoud L. D., Essaqui A., Zertoubi M., Azzi M., Benaissa M. (2014). Study of antioxidant activity and anticorrosion action of the methanol extract of dwarf palm leaves (*Chamaerops humilis* L.) from Morocco. *Journal of Materials Environmental Science*.

[37] Yahyaoui M., Ghazouani N., Sifaoui I., Abderrabba M. (2017). Comparison of the effect of various extraction methods on the phytochemical composition and antioxidant activity of *Thymelaea hirsuta* L. aerial parts in Tunisia. *Biosciences, Biotechnology Research Asia*.

[38] Yahyaoui M., Bouajila J., Cazaux S., Abderrabba M. (2018). The impact of regional locality on chemical composition, anti-oxidant and biological activities of *Thymelaea hirsuta* L. extracts. *Phytomedicine*.

[39] Benhammou N., Ghambaza N., Benabdelkader S., Atik-Bekkara F., Kadifkova P. T. (2013). Phytochemicals and antioxidant properties of extracts from the root and stems of *Anabasis articulata*. *International Food Research Journal*.

[40] Bentabet N., Boucherit-Otmani Z., Boucherit Kebirm Ghaffour K. (2014). Preliminary phytochemical study of leaves and roots of *Fredolia aretioides*, endemic plant of Algeria. *Der Pharma Chemica*.

[41] Ladoh Y., Dibong S., Nyegue M. (2014). Antioxidant activity of methanolic extracts of Phragmanthera capitata (Loranthaceae) collected from *Citrus sinensis*. *Journal of Applied Biosciences*.

[42] Bourgou S., Serairi Beji R., Medini F., Ksouri R. (2016). Effect of the solvent and extraction method on the phenolic compound content and antioxidant potential of *Euphorbia helioscopia*. *Journal of New Sciences, Agriculture and Biotechnology*.

[43] Rhaz N., Oumam M., Hannache H. (2015). Comparison of the impact of different extraction methods on polyphenols yields and tannins extracted from Moroccan *Acacia mollissima* barks. *Industrial Crops and Products*.

[44] Bentabet N. (2014). Phytochemical study and evaluation of the biological activities of two plants *Fredolia aretioides* and *Echium vulgare* from western Algeria [Doctoral thesis in Cell Biology and Biochemistry].

[45] Ozen T., Telci I., Gul F., Demirtas I. (2018). A comprehensive study on phytochemical contents, isolation and antioxidant capacities in wild mind, *Mentha longifolia* subsp. *typhoides* var. *typhoides*. *Morocan Journal of Chemistry*.

[46] Bozin B., Mimica-Dukic N., Samojlik I., Goran A., Igic R. (2008). Phenolic as antioxydants in garlic (*Allium sativum* L. Alliaceae). *Food Chemistry*.

[47] Shakeri A., Hazeri N., Vlizadeh J., Ghasemi A., Tavallaei F. Z. (2012). Phytochemical screening, antimicrobial and antioxidant activities of *Anabasis aphylla* L. extracts. *Kragujevac Journal Science*.

[48] Cai Y., Luo Q., Sun M., Corke H. (2004). Antioxidant activity and phenolic compounds of 112 traditional Chinese medicinal plants associated with anticancer. *Life Sciences*.

[49] Ouedraogo R. A., Koala M., Dabire C. (2015). Total phenol content and antioxidant activity of extracts of the three main varieties of onions (*Allium cepa* L.) grown in the North Central region of Burkina Faso. *International Journal of Biological and Chemical Sciences*.

[50] Okou O. C., Yapo S. E.-S., Kporou K. E., Baibo E. L., Monthaut S., Djaman A. J. (2018). Évaluation de l’activité antibactérienne des extraits de feuilles de *Solanum torvum* Swartz (Solanaceae) sur la croissance in vitro de 3 souches d’entérobactéries. *Journal of Applied Biosciences*.

[51] Prior R. L., Wu X., Schaich K. (2005). Standardized methods for the determination of antioxidant capacity and phenolics in foods and dietary supplements. *Journal of Agricultural and Food Chemistry*.

[52] Teow C. C., Truong V.-D., McFeeters R. F., Thompson R. L., Pecota K. V., Yencho G. C. (2007). Antioxidant activities, phenolic and *β*-carotene contents of sweet potato genotypes with varying flesh colours. *Food Chemistry*.

[53] Floegel A., Kim D.-O., Chung S.-J., Koo S. I., Chun O. K. (2011). Comparison of ABTS/DPPH assays to measure antioxidant capacity in popular antioxidant-rich US foods. *Journal of Food Composition and Analysis*.

[54] Belhadj Tahar S., Hadj-Mahammed M., Yousfi M. (2015). Study of the antioxidant activity of phenolic extracts of *Atriplex halimus* L and Haloxylon scoparium pomel from the northern Sahara. *Annales des Sciences et Technologie*.

[55] Ferreira I. C. F. R., Baptista P., Vilas-BOAS M., Barros L. (2007). Free-radical scavenging capacity and reducing power of wild edible mushrooms from northeast Portugal: individual cap and stipe activity. *Food Chemistry*.

[56] Liu J., Jia L., Kan J., Jin C. (2013). *In vitro* and *in vivo* antioxidant activity of ethanolic extract of white button mushroom (*Agaricus bisporus*). *Food and Chemical Toxicology*.

[57] Bansemir A., Blume M., Schröder S., Lindequist U. (2006). Screening of cultivated seaweeds for antibacterial activity against fish pathogenic bacteria. *Aquaculture*.

[58] Cowan M. M. (1999). Plant products as antimicrobial agents. *Clinical Microbiology Reviews*.

[59] Okou O. C., Yapo S. E.-S., Kporou K. E., Baibo G. L., Monthaut S., Djaman A. J. (2018). Evaluation of the antibacterial activity of *Solanum torvum* Swartz (Solanaceae) leaf extracts on the in vitro growth of 3 strains of enterobacteriaceae. *Journal of Applied Biosciences*.

[60] El-haci I. A. (2014). Phytochemical study and biological activities of some endemic medicinal plants of the Sude of Algeria: ammodaucus leucotrichus Coss. & Dur., Anabasis aretioides Moq. Coss. and Limoniastrum feei (Girard) Batt [Doctoral thesis in Biochemistry].

[61] Amri O., Elguiche R., Tahrouch S., Zekhnini A., Hatimi A. (2015). Antifungal and antioxidant activities of some aromatic and medicinal plants from the southwest of Morocco. *Journal of Chemcal Pharmaceutical Research*.

[62] Guettaf S., Abidli N., Kariche S., Bellebcir L., Bouriche H. (2016). Phytochemical screening and antioxidant activity of aqueous extract of *Genista Saharae (Coss. & Dur.)*. *Der Pharmacia Lettre*.

[63] Kang D. G., Yun C. k., Lee H. S. (2003). Screening and comparison of antioxidant activity of solvent extracts of herbal medicines used in Korea. *Journal of Ethnopharmacology*.

[64] Penchev P. I. (2010). Study of processes for extracting and purifying bioactive products from plants by coupling separative techniques at low and high pressures [Doctoral thesis in Process and Environmental Engineering].

